# Body Pose Estimation Integrated With Notational Analysis: A New Approach to Analyze Penalty Kicks Strategy in Elite Football

**DOI:** 10.3389/fspor.2022.818556

**Published:** 2022-03-10

**Authors:** Guilherme de Sousa Pinheiro, Xing Jin, Varley Teoldo Da Costa, Martin Lames

**Affiliations:** ^1^Performance Analysis and Sports Informatics, Technical University, Munich, Germany; ^2^Department of Informatics, Technical University of Munich, Munich, Germany; ^3^UFMG Soccer Science Center, Department of Sports Science, Federal University of Minas Gerais, Belo Horizonte, Brazil

**Keywords:** body orientation, performance analysis, OSPAF, OpenPose, human movement, motion capture, soccer analytics

## Abstract

Body orientation of football players has proven to be an informative resource related to successful penalty kicks. OpenPose is one of the most popular open-source pose estimation technologies. This study aims: (i) to verify whether OpenPose can detect relevant body orientation angles from video data of penalty kicks in elite football and (ii) to investigate the relationship between these body angles and observable behaviors analyzed *via* an observational system for penalty kick analysis in football (OSPAF) with the penalty taker and goalkeeper strategy. A total of 34 penalty videos, with standardized viewing angle, from the main European leagues (2017–2020) were analyzed. Relevant body orientation variables were selected for penalty kicks analysis and were extracted from video data through OpenPose technique. The OSPAF, previously validated by experts, was used. The mean confidence score of OpenPose measures was 0.80 ± 0.14. OpenPose Retest reliability values was 0.976 ± 0.03. Logistic regressions were performed to investigate the relationship between OpenPose investigated variables (penalty taker: shoulder, hips, and nonkicking foot orientation; goalkeeper: right and left foot, anticipation), observable behaviors (OSPAF variables), and the strategy (penalty taker: goalkeeper dependent or independent; goalkeeper: shooter dependent or independent) in penalty kicks. The selected body orientation angle (goalkeeper anticipation) measured through OpenPose correlated significantly with the goalkeeper strategy. The prediction model of the goalkeeper's strategy had its accuracy increased to 97% when the variable goalkeeper anticipation was included [$\chi_{\left(35\right)}^{2}$ = 49.648, *p* < 0.001]. Lower degrees of goalkeeper anticipation, the goalkeeper tactical action (awaiting), and run up speed (slow) were associated with a kicker-dependent strategy. Regarding the penalty taker, the selected body angles measured through OpenPose did not associate significantly with the shooter strategy. Body orientation analysis by using OpenPose has shown sufficient reliability and provides practical applications for analyzing the strategies adopted by goalkeepers in penalty kicks in elite football.

## Introduction

The analysis of penalty kick performance in football has played an important role in sports analytics (Paterson et al., 2020; Noël et al., 2021; Pinheiro et al., 2021a,b). Over the past 30 years, there have been several scientific studies that identify the motivational-, strategic-, anticipatory-, attention-, and perception-based factors that can mean a successful or failed penalty kick (Memmert and Nöel, 2020). Recent research focusing on the technical dynamics of penalty kicks has also identified multiple key variables that can differentiate the players strategy (Pinheiro et al., 2021b) and enhance the overall chances of scoring a penalty kick (Jamil et al., 2020). The importance of the optimal performance of both the rival players during the penalty kick is paramount, especially since the introduction of the penalty shoot-out in major competitions to determine which team progresses after a drawn match (Fariña et al., 2013).

One prerequisite to increase the probability of successful performance is the implementation of the suitable penalty kick strategy (van der Kamp, 2006). Previous research has identified two main strategies for taking a penalty (Kuhn, 1988; van der Kamp, 2006). First, the keeper-independent strategy, where the kicker selects the target location to shoot toward before the run-up and does not attend to the actions made by the goalkeeper during the run-up. The decision of where to aim depends on the penalty taker's kicking preference (Noël et al., 2015). On the contrary, in the keeper-dependent strategy, the kicker tries to obtain information from the goalkeeper's reactions during the run-up. Nevertheless, the outcome of a penalty is determined by an interaction between the shooter's strategy (e.g., technique, speed) and the goalkeeper's strategy (Hunter et al., 2018; Pinheiro et al., 2021b). The optimal strategy depends on the keeper's behavior and the relative benefits of speed, accuracy, and unpredictability within each situation. Regarding the goalkeeper strategy, there are two approaches: the dependent and independent penalty takers. The goalkeeper who behaves according to the first group defines his movement based on the actions of the penalty taker. The second type of goalkeeper is the one who risks jumping to a corner independently of the kicker's movement (Kuhn, 1988).

The analysis of the penalty kick strategies has been investigated about numerous factors (Noël et al., 2015; Pinheiro et al., 2021b). Noël et al. (2015) developed a method for investigating penalty taker strategies, based on a controlled simulated situation. In a noncompetitive setting, youth players were instructed to take penalty kicks adopting either a keeper-independent or keeper-dependent strategy. Based on this setting, an observational system was developed to evaluate penalty kick performances by using video footage from competitive matches. Those authors identified that attention to the goalkeeper, run-up fluency, and kicking technique in combination could predict kick strategy in 92% of the penalties. However, one possible limitation is that the penalty takers followed a script denoting whether they use a keeper-independent or keeper-dependent strategy and, therefore, the design created differed very importantly from the match situation (Pinheiro et al., 2021a). Besides that, it remains unclear whether the young players disposed of a sufficient skill level to execute both the strategies with the same quality. To address the interaction process in professional football and provide a valid instrument, (Pinheiro et al., 2021b) developed an observational system for penalty kick analysis in football (OSPAF). The OSPAF met all the requirements of instrument validation.

Body orientation has been indicated as a key factor under covering the success in penalty kicks (Li et al., 2015). However, it is a yet little explored area in penalty kick analytics. There is a need within human movement sciences for a markerless motion capture system, which is easy to use and sufficiently accurate to evaluate motor performance (Nakano et al., 2020). OpenPose method adopts unique top-down position recognition by using deep learning and also the unique algorithm as affiliation recognition of body parts by Part Affinity Fields (PAFs) to detect the two-dimensional (2D) pose of multiple people in images (Nakai et al., 2019). OpenPose can recognize skeletons of multiple players in real-time, by using a simple web camera. Given a video or image, OpenPose estimates a total of 25 biometric human body parts (e.g., right knee, left knee, and right foot). The output of the algorithm is in the form of 25 × 3 vector for each individual, where the first two columns of the vector stand for the x-y coordinate of key points in the field domain, while the third column represents the confidence score. This method has shown high-level accuracy on multiple public benchmarks, being efficient for multiperson pose estimation (Cao et al., 2017). Zago et al. (2020) confirmed the feasibility of tracking kinematics by using OpenPose. OpenPose-based markerless motion capture can be used for human movement science with an accuracy of 30 mm or less (Nakano et al., 2020). Despite several studies in this area, key gaps remain, including a lack of research by using OpenPose to detect relevant body orientation angles in field settings and based on sports broadcasts such as penalty kicks from TV videos.

Sangüesa et al. (2019) had previously applied OpenPose to estimate the body orientation of football players from video data during match play. Those authors indicated that a time-based set of player orientations might detect specific situations where orientation is crucial in the match. Recently, Sangüesa et al. (2020a) used a player's body orientation to model pass feasibility in football. The inclusion of the orientation data estimated directly from video frames by using pose models, into a passing model, has proved to be a key feature in the decision-making process of players and is strictly correlated to the play outcome. In another study, Sangüesa et al. (2020b) mapped body pose parts (e.g., shoulders and hips) in a 2D field by combining OpenPose with a super-resolution network and merging the obtained estimation with contextual information (ball position). Results have been validated with players held electronic performance and tracking systems devices, obtaining a median error of 27° per player.

Notation analysis has been widely used to examine the technical properties of football performance through recording behavior incidence (Lames and Hansen, 2001; Hughes and Bartlett, 2004; Sarmento et al., 2014; Casal et al., 2017; Pinheiro et al., 2021b). In the recent years, there has been a vertiginous evolution in the match analysis methods, mainly motivated by the emergence of automatic registration procedures, which allows the immediate acquisition of a large amount of data related to the positioning of the players with the game (Castellano et al., 2014). The rise of sports analytics has provided a new set of metrics and statistics that can serve coaches to evaluate the player (Sangüesa et al., 2019). Nevertheless, one limitation is that one method does not entirely supply all the necessary information. There is, therefore, a need to use multimethod approach to solve sports analytics problems, analyzing variables by using different methods (Aranda et al., 2019). Methodology designs that combine different study approaches (e.g., observational and biomechanical/method that produce body angles), also known as mixed methods (Preciado et al., 2019), tend to provide a deeper understanding and reliability of the studied phenomenon (i.e., penalty kicks).

The influence variables on penalty kicks success are extensively studied (Jamil et al., 2020; Memmert and Nöel, 2020; Paterson et al., 2020; Noël et al., 2021; Pinheiro et al., 2021b). (Pinheiro et al., 2021b) recommended that future studies could use the OSPAF, applying technological methods to analyze its variables, such as computer techniques for body pose estimation and machine learning-based video analysis. To the best of our knowledge, no study has used OpenPose to detect relevant body orientation angles in penalty kicks in elite football from TV broadcast. Therefore, the aims of this study are: (i) to verify whether OpenPose can detect relevant body orientation angles from video data of penalty kicks in elite football and (ii) to investigate the relationship between these body angles and observable behaviors analyzed via OSPAF (Pinheiro et al., 2021b) with the penalty taker and goalkeeper strategy.

## Materials and Methods

### Sample

The dataset consists of 34 penalty kicks from the main European football leagues (Premier League, Ligue 1, Bundesliga, LaLiga, Serie A, and Champions League; seasons 2017–2020). The videos were recorded from TV broadcasters and were registered and analyzed postevent. As the video recordings were public, confidentiality was not an issue and authorization was not required from the players observed or their representatives. The procedures performed in this study were in strict accordance with the Declaration of Helsinki as well as with the ethical standards of the Technical University of Munich.

### Methodological Design

**Figure 1:**
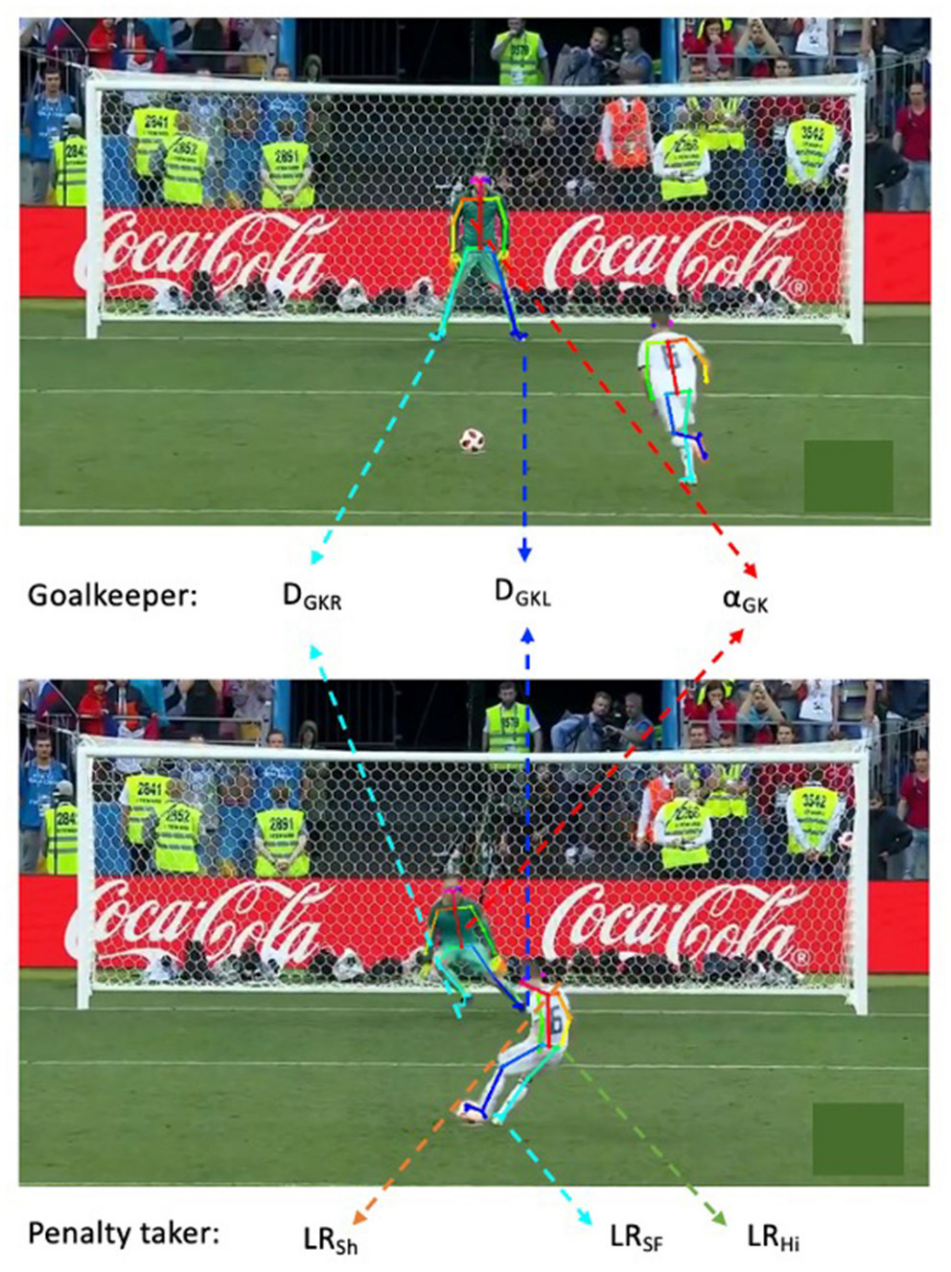
Penalty kick viewing angle, frames analyzed, and process of pose estimation. The upper image corresponds to the moment when the penalty taker starts the run-up approaching the ball, and the down one corresponds to the moment when he touches the ball. The coordinates belonging to shoulders, hips, and non-kicking foot of penalty taker are mapped in a 2D field. LR-side Booleans (LR_Sh_, LR_Hi_, LR_SF_: penalty kicker's shoulder, hips, and non-kicking foot orientation, respectively), angles (α_Sh_ α_Hi_, α_SF_: penalty kicker's shoulder, hip, and non-kicking foot, respectively) and confidences (C_Sh_, C_Hi_, C_SF_: confidence score of the orientation of penalty kicker's shoulder, hips and non-kicking foot, respectively). Coordinates belonging to the neck and hip of the goalkeeper at these two moments are mapped to a vector and the angle is calculated (α_GK_). D_GKR_ and D_GKL_, the movement distance of right and left foot; C_GK_, represent confidence score.

All the penalty kick data were annotated by the researchers with the OSPAF (Pinheiro et al., 2021b). Body orientation was analyzed by using OpenPose (CMU-Perceptual-Computing-Lab, 2017). The choice and analysis of the penalty kick video viewing angle was standardized (Pinheiro et al., 2021b), with a pixel resolution of 1,280 × 720. The viewing angle used in this study was the view behind the penalty taker (Figure 1). The confidence score, calculated by OpenPose, was used to evaluate reliability (Sangüesa et al., 2019). In order to check the stability within the observation, every penalty kick was analyzed with OpenPose twice. Retest reliability was utilized to check these repeated measurements.

### Body Pose Detection and Orientation

OpenPose (version 1.4.0) was installed from GitHub (CMU-Perceptual-Computing-Lab, 2017) and run with a notebook (Apple's M1 Chip) under default settings. Orientation from pose used pretrained models and three-dimensional (3D) vision techniques to obtain a first orientation estimation of each player. Once the pose is extracted for each player, the coordinates and confidence level associated with the body parts are stored to estimate the pose orientation. As a result, in the moving skeletal pictures generated by OpenPose, the skeleton marks are shown and overlapped well with the figure of players (Nakai et al., 2019). For technical details of pose models, see Ramakrishna et al. (2014), Wei et al. (2016), and Cao et al. (2017).

In this study, the orientation of a player's body was defined as the 2D rotation of the player's upper torso around the vertical axis, which is assumed to coincide with the field projection of a normal vector placed in the center of their upper torso, involving both the shoulders and hip parts (Sangüesa et al., 2019). Especially in the case of the non-kicking foot, the hallux and the fifth toe of the support foot were used as the left-right (LR) pair to find the normal vector. Orientation was measured in degrees. For technical details of this methodological approach, see Sangüesa et al. (2019, 2020a,b).

In this study, two frames were analyzed. First, when the penalty taker starts the run-up into the ball and, second, when he touches the ball (Figure 1). Then, the target variables for the penalty taker (nonkick foot orientation, hips, and shoulders) and the goalkeeper [anticipation movement (explained in detail below) and right and left foot orientation] were extracted. There might be blurry frames and overlap of players. OpenPose could then fail to detect the main biometric body parts of the two players involved in this analysis; therefore, in this case, the neighboring frames, in which biometric body parts can be detected, were used.

Once the pose was extracted for the goalkeeper and penalty taker, the direct linear transformation (DLT) algorithm (Hartley and Zisserman, 2004) was used to map the coordinate information of players into a 2D field with a homography, given the 4 field corners' coordinates in the image (or its projection out of the image in the nonvisible cases). The homography was first calculated based on four 2D-to-2D point correspondences between the frames (Equation 1). From the output of OpenPose, the coordinates of the main upper-torso parts are found in the image domain; by mapping the LR pair (either shoulders or hips) in the 2D field, a first insight of the player orientation is obtained. The player can be inclined toward the right (0–90 and 270–360°) or the left (90–270°) side of the field.


(1) $$\begin{array}{r} \left[\begin{array}{c} x^{\prime }\\ y^{\prime }\\ 1 \end{array}\right]=\alpha H\left[\begin{array}{c} x\\ y\\ w \end{array}\right]\;,where\;homography\;H=\left[\begin{array}{c} h_{1}h_{2}h_{3}\\ h_{4}h_{5}h_{6}\\ h_{7}h_{8}h_{9} \end{array}\right] \end{array}$$

After that, the 2D field projections of the LR pair of penalty taker's shoulders, hips, and nonkicking foot (big toe and small toe) were calculated. All the body parts' orientations could point to the left or right half, based on the angle system presented by Sangüesa et al. (2019, 2020a,b). Based on the 2D projection, LR-side Booleans (LR_Sh_, LR_Hi_, and LR_SF_: penalty kicker's shoulder, hips, and non-kicking foot orientation, respectively), angles (α_Sh_ α_Hi_, and α_SF_: penalty kicker's shoulder, hip, and non-kicking foot, respectively), and confidences (C_Sh_, C_Hi_, and C_SF_: confidence score of the orientation of penalty kicker's shoulder, hip, and non-kicking foot, respectively) were obtained. The corresponding confidences are the average of OpenPose's player toes, shoulders, and hips confidences, respectively. Figure 1 shows the output of OpenPose on which the key biometric body parts of an individual are detected, illustrating the estimation process of orientation.

### Anticipation Movement of the Goalkeeper

The anticipation movement of the goalkeeper in the penalty kick was defined here as to how far the goalkeeper moves between: (1) the moment when the penalty taker starts the run-up approaching the ball and the (2) moment when the penalty taker first touches the ball. In detail, the line formed by the connection between the goalkeeper's neck and the middle of the hip was used to depict the position status of the goalkeeper in these two moments. Furthermore, the angle (αGK) between the two lines drawn from the two moments measures the anticipation movement of the goalkeeper. The confidence level for this measure (C_GK_) was calculated by the average confidence scores of the neck and middle of the hip. This process is given in Figure 1.

The movement distance of the goalkeeper's left and right foot was also used to measure the anticipation movement. Left and right ankles were used to represent the left and right feet, respectively; moreover, coordinate information together with metric Euclidean distance was used to depict the movement distance of the goalkeeper's feet, as shown in Figure 1.

### Ball Speed

Ball speed was determined with the open-source software program Kinovea motion analysis (version 0.8.15, Kinovea, France). This software has already been used in various studies analyzing penalty kicks (Hunter et al., 2018; Makaruk et al., 2019).

### Notational Analysis

A previously developed and validated observational system (OSPAF) for penalty analysis in elite football was also used in this study (Pinheiro et al., 2021b). The protocols for the use of observational systems were adopted (Lames and Hansen, 2001; Aranda et al., 2019; Fernandes et al., 2019). All the observable behaviors recorded are shown in Table 1.

**Table 1:** OSPAF variables.

**Variables**	**Definition**	**Attribute levels**
Run up speed	Running speed of the penalty kicker toward the ball	Fast or slow
Run up fluency	Characteristic of the penalty kicker's run during the approach of the ball, with or without pauses.	Continuous running or running with pauses
Run up approach angle	Penalty kicker's running angle to the ball.	Frontal or diagonal
Number of steps	Number of steps of the penalty kicker until contact with the ball	1–3; 3–5; or +5
Kicking technique	The technique used by the penalty kicker to kick the ball	Side foot kick or instep kick
Foot used to kick	Foot used by the penalty kicker to kick the ball	Right or left
Penalty taker gaze behavior	Gaze behavior of the kicker during the approach run.	Gaze at the ball or not at the ball
Goalkeeper (GK) initial posture	Position of the body segments.	Arms raised; arms down or arms extended in a position perpendicular to the goalkeeper 's trunk
Deception by the penalty taker	Indication if the kicker has done any action to distract the goalkeeper during his or her run-up	Yes or no
Goalkeeper tactical action	General evaluation of the way the goalkeeper acted during the penalty shoot-out, to the anticipatory aspect	Try to guess the location of the shot; or awaiting the penalty taker action
Goalkeeper performance	Evaluation of the goalkeeper's performance according to his movement and contact with the ball	0: GK made any final movement to the side of the goal opposite to the final ball location; 1: GK did not move from the center of the goal; 2: GK made a movement in the correct direction but did not dive and failed to make contact with the ball; 3: GK dived in the correct direction but failed to make contact with the ball; 4: GK dived in the correct direction and contacted the ball without saving it; or 5: GK successfully saved the kick
Moment of the match	Time of the match when the penalty will be taken	First half; second half or extra time or shoot out
Location of the match (kicker point of view)	Indication if the penalty kicker is from the home team, visitor, or if he plays on a neutral field.	Home, neutral or away
Momentary result (kicker point of view)	Result of the match (for the penalty kicker) at the moment the penalty was marked.	Winning, drawing or losing
Momentary result (GK point of view)	Result of the match (for the Goalkeeper) at the moment the penalty was marked.	Winning, drawing or losing
Match importance	Level of importance of the match for the team	Championship final match; decisive knockout match; group stage match; early season game; match in final stages of the season
Penalty kick direction	The direction of the ball on goal	Left; center or right
Penalty kick height	Height of the ball on goal	Upper; center or down
Penalty kick outcome	Result of the penalty kick	Goal; saved by goalkeeper or Shot misses goal (wide, over or post)
Penalty taker strategy	Overall strategy perceived by the observer (6)	Goalkeeper dependent; unclear or goalkeeper independent
Goalkeeper strategy	Overall strategy perceived by the observer (6)	Kicker independent; unclear or kicker dependent

### Data Analysis

**Figure 2:**
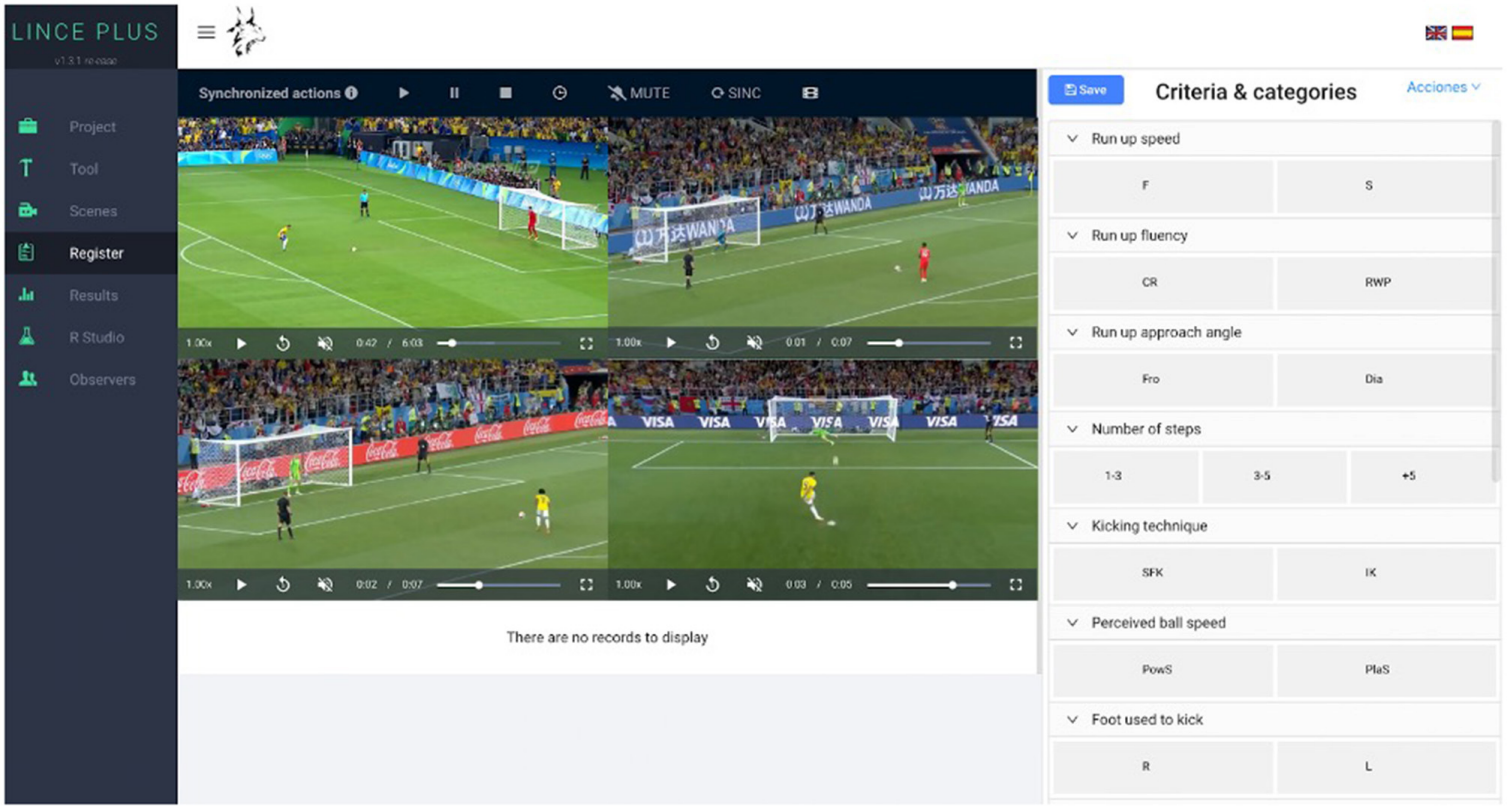
Lince Plus interface. Example of criteria and categories coding.

For descriptive analysis, mean and SD were used. The Shapiro–Wilk test was performed to verify data normality. The association level between the OSPAF variables with the penalty taker and goalkeeper strategy was determined with the use of the chi-squared (χ^2^) test. The effect size was determined by using the Cramer's V and classified as weak (ES ≤ 0.2), moderate (0.2 < ES ≤ 0.6), and strong (ES > 0.6) (Cohen, 1988). The association level between OpenPose variables with the penalty taker and goalkeeper strategy was determined with the use of the point-biserial correlation. Retest reliability was utilized to check the repeated measurements of OpenPose (Vilagut, 2014). Test-retest reliability coefficients (also called coefficients of stability) vary between 0 and 1, where 1: perfect reliability, ≥ 0.9: excellent reliability, ≥ 0.8 < 0.9: good reliability, ≥ 0.7 < 0.8: acceptable reliability, ≥ 0.6 < 0.7: questionable reliability, ≥ 0.5 < 0.6: poor reliability, < 0.5: unacceptable reliability, and 0: no reliability (Vogt, 2005; Lindstrom, 2010). To identify which variables would be able to predict the penalty takers and goalkeeper strategy, the logistic regression (*enter method*) analyses were performed. Dimensions and categories of OSPAF were coded in Lince software (Figure 2; Gabin et al., 2012; Soto et al., 2019). Kappa levels of the OSPAF were 0.90 and 0.86—intra- and interreliability (Pinheiro et al., 2021b). The interpretation of this coefficient was adopted as follows: κ > 0.8: very good; 0.6 < κ < 0.8: good; 0.4 < κ < 0.6: moderate; 0.2 < κ < 0.4: fair; and κ < 0.2: poor (Altman, 1991; O'Donoghue, 2010). The level of statistical significance adopted was α = 0.05, with a 95% CI. All the data were analyzed by using JASP software (JASP Team, 2021; Computer software; JASP Version 0.14).

## Results

Descriptive data of all the OpenPose and OSPAF variables analyzed are presented as Supplementary Material.

### OpenPose Confidence Score and Retest Reliability

The mean confidence score of OpenPose measures was 0.80 ± 0.14. The confidence score per variable is shown in Table 2.

**Table 2:** OpenPose confidence score per variable.

**Player**	**Body orientation angle**	**Confidence score**
Penalty taker	Non-kick foot orientation	0.51
	Shoulders	0.87
	Hips	0.85
Goalkeeper	Anticipation	0.87
	Left foot	0.84
	Right foot	0.83

Test-retest reliability values are shown in Table 3.

**Table 3:** Test-retest reliability per variable.

**Player**	**Body orientation angle**	***r***
Penalty taker	Non-kick foot orientation	0.924^*^
	Shoulders	0.998^*^
	Hips	0.991^*^
Goalkeeper	Anticipation	0.998^*^
	Left foot	0.953^*^
	Right foot	0.961^*^

* *p < 0.05*.

### Influence Variables on Goalkeeper Strategy

The association between all the OpenPose and OSPAF variables with the goalkeeper's strategy was analyzed. Table 4 presents only the variables that presented association and the respective values.

**Table 4:** Association between OSPAF and OpenPose variables with the goalkeeper strategy.

	**OSPAF variables**	**χ^2^**	***p***	**Cramer's V**
Goalkeeper strategy	Run up speed	4.875	<0.05	0.354
	GK tactical action	26.542	<0.05	0.825
	**OpenPose variable**	*r*_**pb**_	***p***	
	Goalkeeper anticipation	0.959	<0.05	

A logistic regression (*enter method*) was performed to investigate the relationship between the goalkeeper's tactical action and run-up speed on the likelihood of the goalkeeper strategy. The logistic regression model was statistically significant, $\chi_{\left(36\right)}^{2}$ = 28.592, *p* < 0.001. The model correctly classified 84.6% of cases. The goalkeeper's tactical action (awaiting) and run speed (slow) were related to a kicker-dependent strategy. While including the correlated OpenPose variable (goalkeeper anticipation) in the model [$\chi_{\left(35\right)}^{2}$ = 49.648, *p* < 0.001], the accuracy is increased to 97.0%. Therefore, lower degrees of goalkeeper anticipation, the goalkeeper tactical action (awaiting), and run-up speed (slow) were associated with a kicker-dependent strategy.

### Influence Variables on Penalty Taker Strategy

The association between all the OSPAF and OpenPose variables with the penalty taker's strategy was analyzed. Table 5 presents only the variables that presented association and the respective values.

**Table 5:** Association between OSPAF and OpenPose variables with the penalty taker strategy.

	**OSPAF variables**	**χ^2^**	***p***	**Cramer's V**
Penalty taker strategy	Run up speed	2.300	<0.05	0.243
	Run up fluency	5.512	<0.05	0.376
	Gaze behavior	22.224	<0.05	0.755
	Deception	8.770	<0.05	0.474
	**OpenPose variable**	*r*_**pb**_	***p***	
	Ball speed	0.927	<0.05	

A logistic regression (*enter method*) was performed to investigate the relationship between the correlated OSPAF variables (run-up speed, run-up fluency, penalty taker gaze behavior, deception by penalty taker, and ball speed) on the likelihood of the goalkeeper-dependent strategy. The logistic regression model was statistically significant, $\chi_{\left(33\right)}^{2}$ = 24.819, *p* < 0.001. The model correctly classified 97.1% of cases. The run-up speed slow, run-up fluency running with pauses, penalty taker gaze behavior not at the ball, the deception performed by the penalty taker, and lower ball speed were related to a goalkeeper-dependent strategy.

## Discussion

A unique method to calculate football players' orientation in in-match penalty kicks from a video has been tested. The mean confidence score of OpenPose variables was 0.80 and test-retest reliability showed an excellent reliability (Vogt, 2005; Lindstrom, 2010). The selected body orientation angle (goalkeeper anticipation) measured through OpenPose correlated significantly with the goalkeeper strategy. The prediction model of the goalkeeper's strategy had its accuracy increased when the variable goalkeeper anticipation was included. This finding corroborates the applicability of OpenPose to obtain the body orientation of professional football players during matches (Sangüesa et al., 2019).

Goalkeepers face a clear trade-off between moving early and moving in the correct direction (Hunter et al., 2018). The goalkeeper's chance of successfully saving a penalty kick is lower than that of the penalty taker to score and he must try to reverse this disadvantage by positioning himself to anticipate the direction of the kick that is about to come (Kuhn, 1988). In this study, the goalkeeper tactical action (awaiting) and run-up speed of the penalty taker (slow) were associated with a kicker-dependent strategy (84.6%). To further improve this model, the inclusion of the correlated OpenPose variable (i.e., goalkeeper anticipation) correctly classified 97.0% of cases. Corroborating previous studies (Nakai et al., 2019; Sangüesa et al., 2019, 2020a,b), the analysis of the body orientation through OpenPose has proved to be extremely useful on penalty kick analytics. The improvement in the model related to the goalkeeper strategy shows the important practical application through the evaluation of the body orientation of football players by using OpenPose as a tool. These findings support previous study by Sangüesa et al. (2019, 2020a,b) and Nakai et al. (2019), which showed that skeletal data recognized by OpenPose are found to be highly applicable with sufficient accuracy. The acquisition of a set of biometric human body part orientations implies an improvement of the analysis of the penalty kick in elite football. Moreover, its integration with video allows this model to be used as a coaching resource to assess players' orientation and improve training strategies for game preparation.

Previous study has shown that the penalty outcome depends, above all, on the emerging results of the “penalty taker—goalkeeper” dyadic interaction (Lopes et al., 2012; Almeida et al., 2016; Pinheiro et al., 2021b). In this study, lower degrees of goalkeeper anticipation, the goalkeeper tactical action (awaiting), and run-up speed of the penalty taker (slow) were associated with a kicker-dependent strategy. From a behavioral perspective, the present findings corroborate this dyadic interaction between the players in a penalty kick, as results showed that the goalkeeper strategy is influenced by the run-up speed of the penalty taker. Corroborating with this finding, Noël et al. (2021) indicated that goalkeepers must consider the penalty taker's run-up for deciding when to initiate their jump to the ball. It is presumed that more successful goalkeepers wait longer to decide for a goal side because this allows them to access more reliable information from the penalty taker's kicking actions to anticipate the penalty takers' intentions (Noël et al., 2021). Analytical procedures that integrate the study of criteria related to the interactions between opponents are highly recommended in game analysis in football (Sarmento et al., 2014). In real competitions, penalty kicks are an interaction process and the observable performance is rather the emergent result of this interaction process than the display of skills and abilities of the two parties (Lames, 2006). The new approach presented in this study, combining different methods, provides a deeper understanding of the player strategy in penalty kicks, through objective identification of the anticipation of the goalkeeper (i.e., angle: αGK measured via OpenPose). To further clarify the process of interaction in the penalty kick and the goalkeeper response time, future studies could introduce a time interval before the kick or an event (exact moment of the kick) as new variables with objective parameters to be analyzed by using OpenPose.

Regarding the penalty taker, the selected body angles measured through OpenPose did not associate significantly with the shooter strategy. A possible explanation could be that the biomechanical patterns of approaching the ball during the kick may vary from player to player, regardless of the strategy adopted. Previous study has shown that kicking from an approach angle of 45 and 60° may alter aspects of kick technique, such as enhancing pelvic rotation and thigh abduction of the kicking leg at impact (Scurr and Hall, 2009). Reinforcing this, Prassas et al. (1990) reported significant differences for a substantial number of variables, related to the kicking foot, leg, the non-kicking foot, trunk, and hip segments in football kicks.

A novelty of this study is the adoption of OpenPose measurements with notational analysis (i.e., OSPAF) to analyze penalty kicks. The OSPAF is an adequate and consistent instrument for analyzing successful and non-successful penalty kick patterns (Pinheiro et al., 2021b). The analysis of observational variables in penalty shooting may provide a general description of its technical execution, which allows for detecting the shooters and the goalkeeper's strategy based on the behavioral variables studied (Pinheiro et al., 2021b). Although the variables used to detect body angles possibly relevant to the analysis of strategy of the shooter in penalty kicks in football did not correlate significantly with the penalty taker strategy, the variables measured by OSPAF (i.e., run-up speed, run-up fluency, penalty taker gaze behavior, deception by penalty taker, and ball speed) were able to correctly classify 97.1% of the penalty taker strategy. The run-up speed slow, run-up fluency running with pauses, penalty taker gaze behavior not at the ball, the deception performed by the penalty taker, and lower ball speed were related to a goalkeeper-dependent strategy. Partially corroborating these findings, Noël et al. (2015) identified three variables (attention to the goalkeeper, run-up fluency, and kicking technique) that in combination could predict kick strategy in 92% of the penalties. Previous study had also shown that run-up and spatiotemporal patterns of gaze may differ between strategies (Noël and van der Kamp, 2012; Noël et al., 2015). The difference in fluency is probably a consequence of penalty takers who use a keeper-dependent strategy to increase time at the end of the run-up by waiting for the goalkeeper to commit to one side of the goal (van der Kamp, 2006). Studies in a realistic setup pointed those penalty takers by using the keeper-dependent strategy direct their gaze more toward the goalkeeper compared to the ball and the target location (Kurz et al., 2018). In contrast, penalty takers by using the keeper-independent strategy direct their gaze more toward the ball compared to the goalkeeper and the target location (Noël and van der Kamp, 2012).

Several studies have investigated the penalty kick strategies in football (van der Kamp, 2006; Noël et al., 2015, 2021; Pinheiro et al., 2021b). However, to the best of our knowledge, this is the first study to use OpenPose to detect relevant body orientation angles in penalty kicks in elite football from TV broadcast. This study is a preliminary study in penalty kick analysis and, thus, requires further examination. This study limitation was to not use a larger sample (e.g., full season), as it could bring practical applications and be more representative. Another limitation of this study was using only one viewing angle. It was included only one standard viewing angle and video quality was standardized, as recommended by (Sangüesa et al., 2020b). Nevertheless, for comparison of penalties from different viewing angles, a 3D transformation must be adopted when using OpenPose. Camera positioning (e.g., viewing angles) could affect the accuracy and, thus, the feasibility of the systems in practical settings (Zago et al., 2020). Nonetheless, this study presents an innovative approach to the analysis of penalty kicks in football, combining notational analysis with OpenPose. Its integration with video specification allows this model to be used as a coaching tool to assess players' orientation under different penalty kicks, improving sports preparation against upcoming opponents.

Multiple practical applications can be provided, from improving and refining player strategy in penalty kicks, to producing a precise assessment of player orientation in high-level competitive scenarios. Although it is not optimal to analyze only 34 penalty kicks, results from the present preliminary data indicate that it is possible to distinguish the goalkeeper's strategy (i.e., kicker dependent vs. kicker independent) based on the degree of goalkeeper anticipation, extracted through OpenPose. The body orientation analysis gives practitioners the potential to quickly evaluate the temporal decision-making of the goalkeeper (i.e., anticipation movement of the goalkeeper) with consideration to choosing when to initiate their jump to the ball. This could help to identify which goalkeepers move early or late in the penalty kick situation. Based on the pattern of anticipation of the goalkeeper in official competitions, specific training strategies can then be developed. Besides, having a time-based set of player orientations enhances analysts' ability to evaluate the relationship of on-ball and off-ball direction with the anatomical patterns. Posture analysis by using OpenPose has been verified to be practical with our model on the goalkeeper strategy identification. Future study could train a deep learning model to provide results about pose orientation automatically and faster.

## Conclusion

This study tested an innovative approach in applying OpenPose measures integrated with notational analysis to investigate the factors influencing the players' strategy in penalty kicks. Results showed the applicability of OpenPose for in-match penalty kick analysis and an improvement in the prediction of the goalkeeper strategy by using a body orientation variable (anticipation) extracted via OpenPose. The goalkeeper degree of anticipation, tactical action, and run-up speed of the penalty taker can be associated with the goalkeeper strategy. Observable variables such as run-up speed, run-up fluency, penalty taker gaze behavior, deception by penalty taker, and ball speed may identify the shooter strategy.

## Data Availability Statement

The original contributions presented in the study are included in the article/Supplementary Material, further inquiries can be directed to the corresponding author/s.

## Ethics Statement

Written informed consent was not obtained from the individual(s) for the publication of any potentially identifiable images or data included in this article. The video recordings used were public, thus authorization was not required from the observed players.

## Author Contributions

GP and ML contributed to the conception, design of this study, and wrote the first draft of the manuscript. GP and XJ organized the database. GP performed the statistical analysis. GP, VC, and ML contributed to the revision of the manuscript and read and approved the presented version of the manuscript. All authors contributed to the article and approved the submitted version.

## Funding

Funding as a doctoral scholarship was provided by the Katholische Akademische Ausländerdienst (KAAD); the publication costs were supported by the Technical University of Munich (TUM) Publishing Fund.

## Conflict of Interest

The authors declare that the research was conducted in the absence of any commercial or financial relationships that could be construed as a potential conflict of interest.

## Publisher's Note

All claims expressed in this article are solely those of the authors and do not necessarily represent those of their affiliated organizations, or those of the publisher, the editors and the reviewers. Any product that may be evaluated in this article, or claim that may be made by its manufacturer, is not guaranteed or endorsed by the publisher.
