# Supplementary material for: Body Pose Estimation Integrated With Notational Analysis: A New Approach to Analyze Penalty Kicks Strategy in Elite Football
Source: Front Sports Act Living. 2022 Mar 10;4:818556. doi: 10.3389/fspor.2022.818556 (PMC8964455; doi:10.3389/fspor.2022.818556)

Supplementary Material

# Supplementary Data

Graph 1. Descriptive data of OpenPose variables


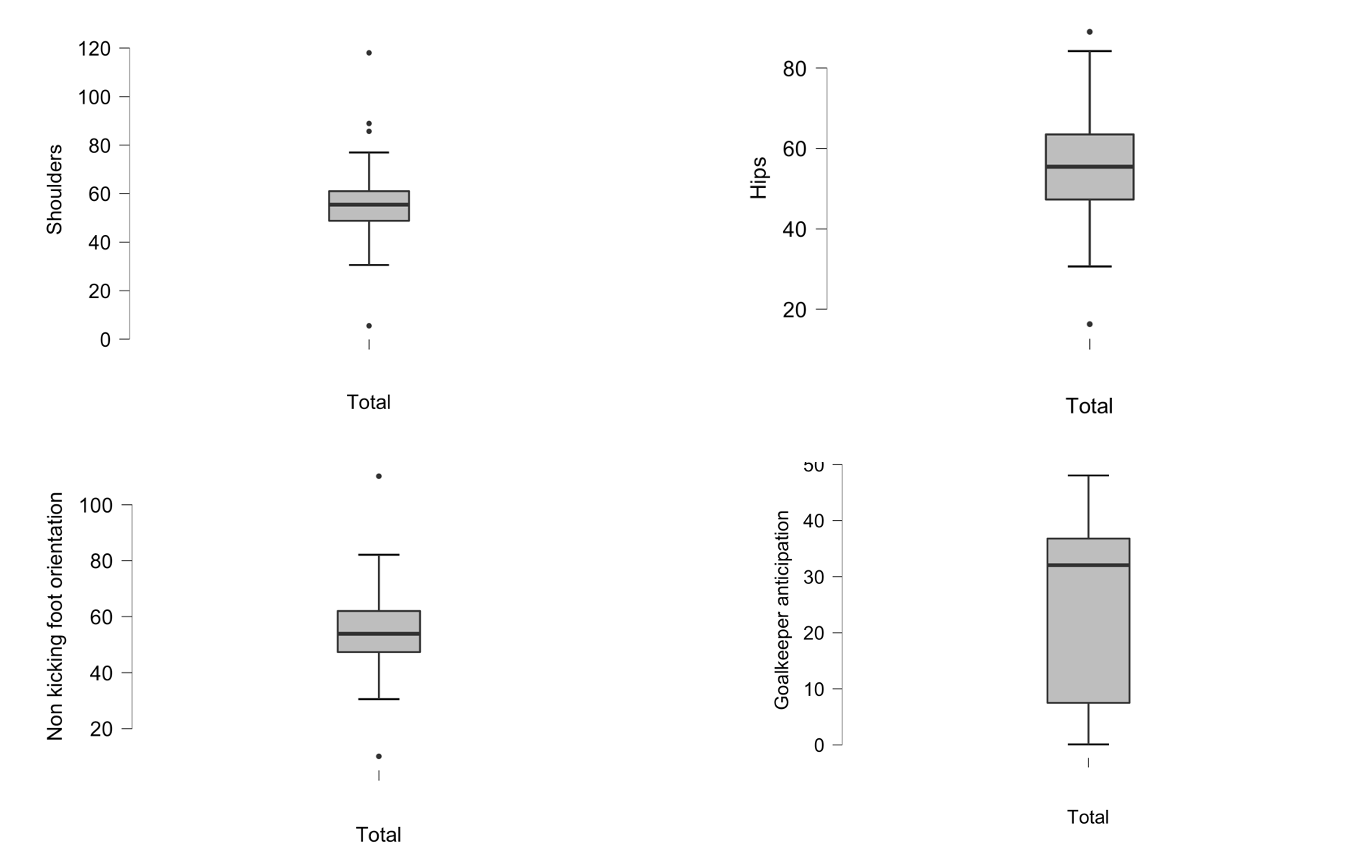


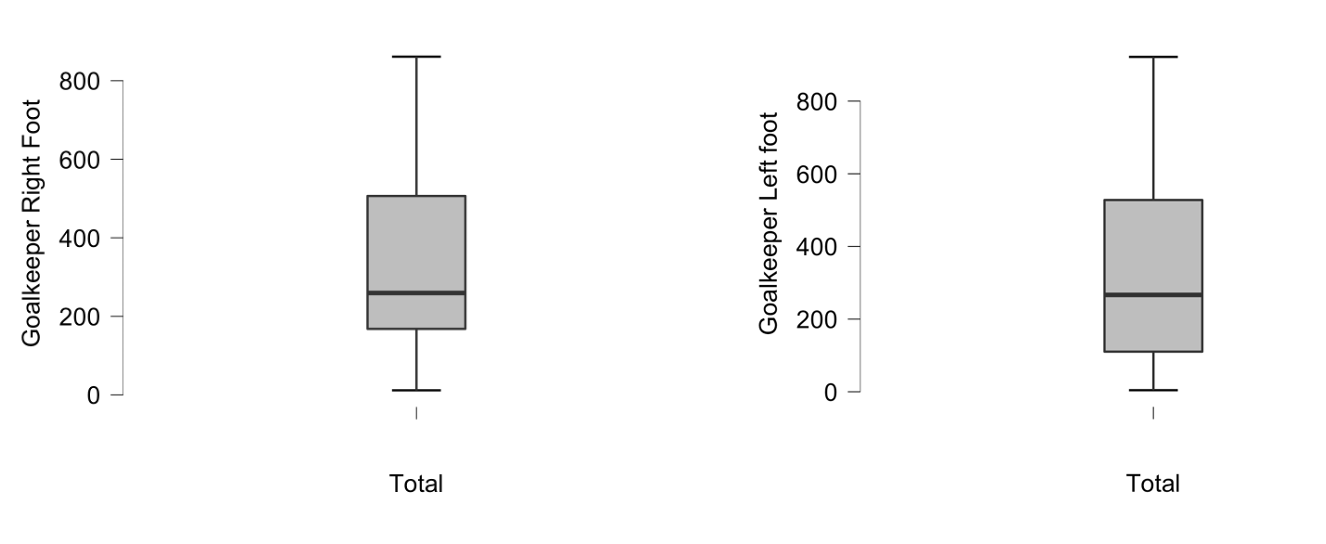


The following boxplots (Graph 2) show the distribution of the OSPAF variables.

Graph 2. Distribution of OSPAF variables


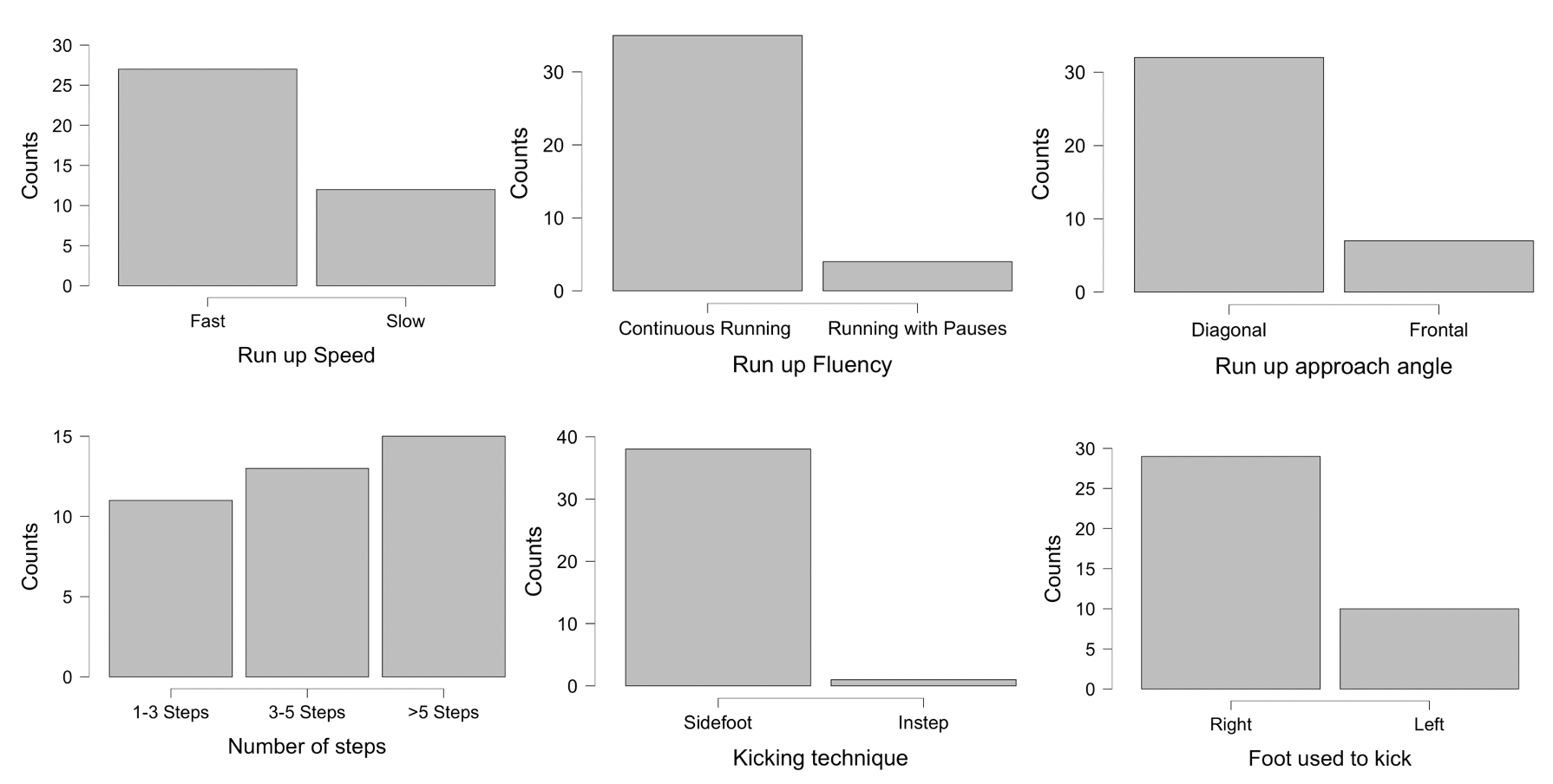


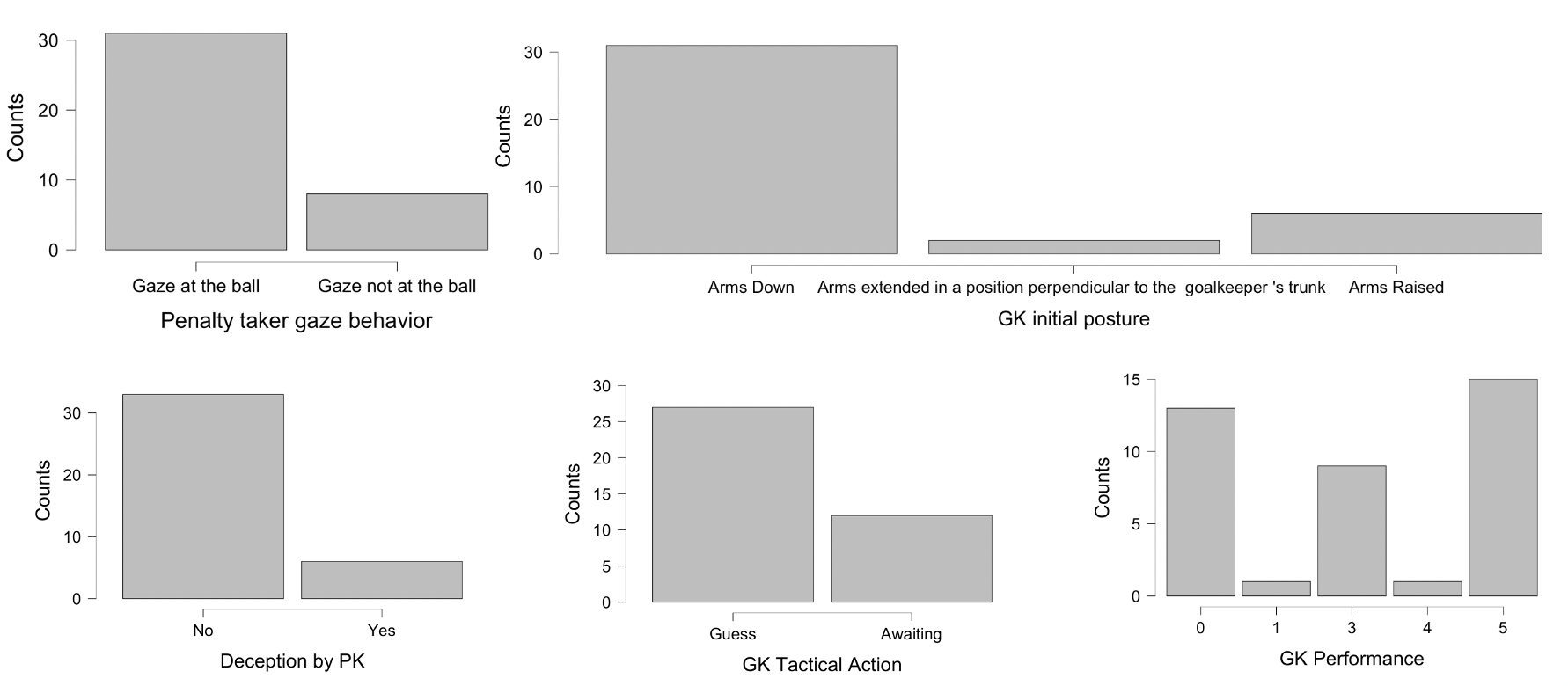

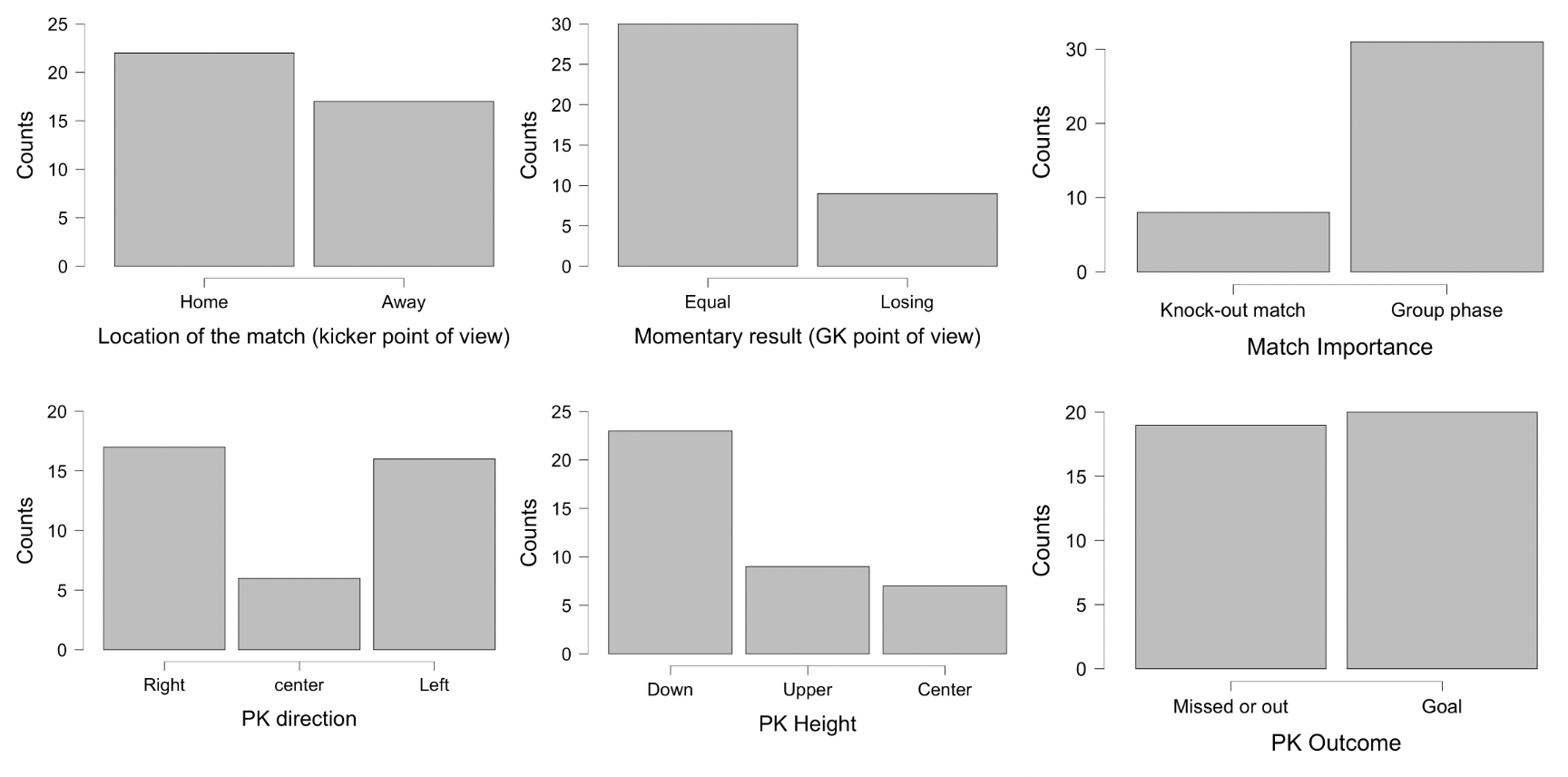


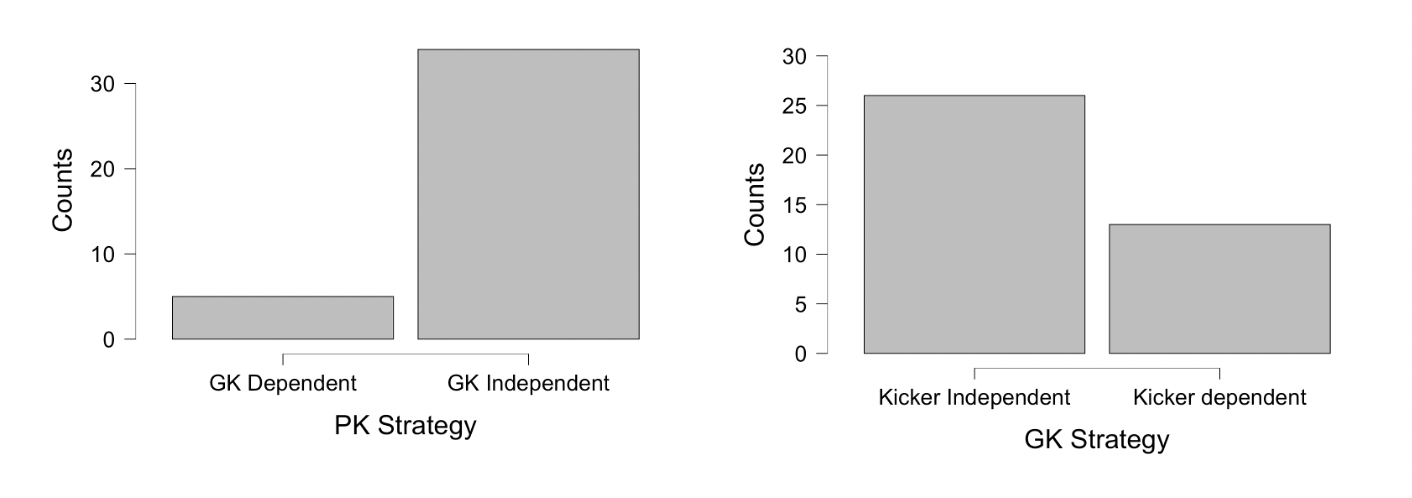

Supplement: Supplementary file 1 [file Data_Sheet_1.docx]
